# Use of Artificial Intelligence in Diagnosing Vertical Root Fractures—A Systematic Review

**DOI:** 10.3390/diagnostics16030406

**Published:** 2026-01-27

**Authors:** Abdulmajeed Saeed Alshahrani, Ahmed Ali Alelyani, Ahmad Jabali, Ahmed Abdullah Al Malwi, Riyadh Alroomy, Amal S. Shaiban, Raid Abdullah Almnea, Vini Mehta, Mohammed M. Al Moaleem

**Affiliations:** 1 Department of Restorative Dentistry, Division of Endodontics, College of Dentistry, Najran University, Najran 61441, Saudi Arabia; musader11@hotmail.com (A.S.A.); aaalelyani1@gmail.com (A.A.A.); 2 Department of Restorative Dental Sciences, College of Dentistry, Jazan University, Jazan 45142, Saudi Arabia; ajabali@jazanu.edu.sa; 3 Department of Restorative Dentistry, Division of Endodontics, College of Dentistry, King Khalid University, Abha 62521, Saudi Arabia; aaalmalwi@kku.edu.sa (A.A.A.M.); ashiban@kku.edu.sa (A.S.S.); 4 Department of Conservative Dental Science, College of Dentistry, Qassim University, Buraydah 52571, Saudi Arabia; r.alroomy@qu.edu.sa; 5 Department of Dental Research Cell, Dr. D. Y. Patil Dental College & Hospital, Dr. D. Y. Patil Vidyapeeth (Deemed to be University), Pimpri, Pune 411018, India; vmehta@statsense.in; 6 Department of Prosthetic Dental Science, College of Dentistry, Jazan University, Jazan 45142, Saudi Arabia; malmoaleem@jazanu.edu.sa

**Keywords:** artificial intelligence, vertical root fractures, periapical radiography, panoramic radiography, cone-beam computed tomography

## Abstract

**Background/Objectives:** Vertical root fractures (VRFs) present significant diagnostic challenges due to their subtle radiographic features and variability across imaging modalities. Artificial intelligence (AI) offers potential to improve detection accuracy, yet evidence regarding its performance across different imaging systems remains fragmented. To critically evaluate current evidence on AI-assisted detection of VRFs across periapical radiography, panoramic radiography, and cone-beam computed tomography (CBCT) and to compare diagnostic performance, methodological strengths, and limitations. **Methods:** A systematic review of literature up to January 2025 was carried out using databases such as PubMed, Scopus, Web of Science, and the Cochrane Library. The studies included in this review utilized AI-based techniques for detecting VRF through periapical, panoramic, or CBCT imaging. Extracted data encompassed study design, AI models, dataset sizes, preprocessing methods, imaging parameters, validation techniques, and diagnostic metrics. The risk of bias in these studies was evaluated using the QUADAS-2 tool. **Results:** Ten studies met inclusion criteria; CNN-based models predominated, with performance highly dependent on imaging modality. CBCT-based AI systems achieved the highest diagnostic accuracy (91.4–97.8%) and specificity (90.7–100%), followed by periapical radiography models with accuracies up to 95.7% in controlled settings. Panoramic radiography models demonstrated lower sensitivity (0.45–0.75) but maintained high precision (0.93) in certain contexts. Most studies reported improvements over human performance, yet limitations included small datasets, heterogeneous methodologies, and risk of overfitting. **Conclusions:** AI-assisted VRF detection shows promising accuracy, particularly with CBCT imaging, but current evidence is constrained by methodological variability and limited clinical validation.

## 1. Introduction

Vertical root fractures (VRFs) are longitudinal structural discontinuities that extend from the root canal space toward the external root surface and adjacent periodontium. These fractures are a serious clinical concern in endodontics, often compromising tooth prognosis and leading to extraction when diagnosis is delayed or missed [1,2]. While VRFs are most commonly found in endodontically treated teeth, they may also occur in vital teeth due to factors such as anatomical variations, occlusal trauma, or iatrogenic procedures [3].

Epidemiological data on VRF prevalence vary significantly depending on diagnostic methodology and patient populations studied [3,4]. Although less frequent in the general population, VRFs remain clinically relevant as they often present with nonspecific symptoms such as localized periodontal issues or persistent periapical pathology in treated teeth [4,5].

The diagnosis of VRFs poses a considerable challenge due to their subtle clinical and radiographic presentation. Clinical signs such as sinus tracts, mild pain, or isolated deep periodontal pockets are nonspecific and may mimic other endodontic or periodontal conditions [4,5]. Conventional periapical radiographs have a limited detection rate for VRFs, particularly when the fracture plane is buccolingually oriented and not aligned with the X-ray beam [6].

Three-dimensional imaging via cone-beam computed tomography (CBCT) has improved diagnostic potential by visualizing root fractures in multiple planes. CBCT can depict signs such as hypodense fracture lines or crestal bone changes suggestive of VRFs [7,8]. However, image artefacts from intracanal materials, beam hardening, and limited resolution remain obstacles to definitive diagnosis, and interpretation remains operator-dependent and subjective [9].

AI is increasingly acknowledged as a revolutionary tool in the fields of medical and dental diagnostics. By utilizing machine learning and deep learning algorithms, AI systems are capable of detecting subtle patterns and features in imaging data that might be overlooked by human observers [10]. In the realm of dentistry, AI has been employed in various areas such as detecting caries, assessing periodontal bone loss, planning orthodontic treatments, screening for oral cancer, and diagnosing endodontic issues [11,12].

The integration of AI in endodontics is particularly relevant for conditions like VRFs, where early and accurate detection significantly influences prognosis. AI systems have the potential to enhance diagnostic consistency, reduce observer bias, and optimize clinical workflows. These technologies can be applied to various imaging modalities—periapical radiographs, panoramic radiographs, and CBCT scans—each with unique advantages and limitations. For VRF detection, AI offers the possibility of combining advanced image processing with predictive modelling to aid clinical decision-making [13,14].

The rationale for exploring AI in VRF detection stems from three primary considerations. First, the morphological characteristics of VRFs often fine and extending longitudinally make them inherently difficult to detect using traditional methods, even with advanced imaging. AI algorithms can be trained to recognize subtle textural, structural, and contextual cues that indicate fracture presence. Second, AI offers the ability to process large volumes of imaging data quickly and consistently, reducing the variability seen between practitioners with different levels of experience. This standardization could be particularly valuable in settings where specialist radiological interpretation is not readily available [11]. Third, AI can potentially integrate multimodal inputs combining radiographic findings with patient demographics, clinical signs, and treatment history to improve diagnostic accuracy and risk stratification [15]. This aligns with the broader movement toward precision dentistry, where individualized patient factors inform diagnosis and management.

Despite the growing interest in AI applications for VRF detection, the existing literature remains fragmented. Studies vary widely in imaging modality, AI architecture, dataset size, and validation methodology [13,16,17,18]. There is also a lack of standardization in performance reporting, with some studies emphasizing sensitivity, others specificity, and others area under the curve (AUC). Furthermore, few studies have addressed how AI systems could be seamlessly integrated into the clinical diagnostic pathway, including considerations of cost-effectiveness, user training, and patient acceptance [18].

Given the heterogeneity of study designs and the rapid evolution of AI methodologies, a systematic synthesis of the literature is warranted. Such a synthesis can identify the most promising imaging modalities and AI approaches, assess the methodological quality of existing research, and highlight key limitations that should be addressed in future studies. Importantly, it can also evaluate the translational potential of AI-assisted VRF detection from laboratory research to routine clinical practice.

This systematic review aims to critically evaluate current evidence on AI-assisted detection of VRFs across various imaging modalities, including periapical radiography, panoramic radiography, and CBCT. The review will compare diagnostic approaches, summarize methodological strengths and weaknesses, and discuss implications for clinical practice and future research.

## 2. Materials and Methods

This systematic review was conducted in accordance with the Preferred Reporting Items for Systematic Reviews and Meta-Analyses (PRISMA) 2020 guidelines to ensure comprehensive and transparent reporting [19] (Table S1). The study protocol was prospectively registered with the International Prospective Register of Systematic Reviews (PROSPERO) under the registration number: CRD420251134312.

### 2.1. Eligibility Criteria

Studies were eligible if they evaluated patients with confirmed VRFs identified through clinical, radiographic, or endoscopic methods, including both endodontically treated and untreated teeth. Inclusion required the use of AI systems, such as deep learning or machine learning, or image processing techniques for VRF detection in dental radiographs, with performance evaluated using validated methods. Studies comparing AI models with human observers, assessing diagnostic accuracy across imaging modalities or tooth types, or analyzing the effect of image enhancement were included. Eligible outcomes comprised quantitative diagnostic performance metrics or assessments of clinical applicability. Only experimental or observational studies published in English through July 2025 with full-text availability were included. Excluded were studies unrelated to VRFs, lacking AI-based methods or validation, without comparison groups, reporting qualitative outcomes only, or designed as reviews, case reports, or narrative articles. The detailed inclusion and exclusion criteria is given in Table S2.

### 2.2. Literature Search

A comprehensive search was conducted across four electronic databases: PubMed, Embase, Web of Science, and Scopus, to identify studies investigating the application of AI in the detection of VRFs in dental imaging. In PubMed, the strategy combined Medical Subject Headings (MeSH), such as Artificial Intelligence and Root Fractures, with related free-text terms using Boolean operators. Embase searches utilized Emtree terms alongside similar keywords, adapted for its indexing structure. In Web of Science, topic field tags (TS) were applied, while Scopus employed TITLE-ABS-KEY fields to capture relevant titles and abstracts. Across all databases, AI-related terms (e.g., “machine learning,” “computer vision”) were combined with fracture-related terms (e.g., “vertical root fracture,” “root cracks,” “dental fractures”) using structured Boolean logic. The search was limited to English-language articles. Additionally, reference lists of all included studies were manually screened to identify other relevant publications. The full search strategy is given in Table S3.

### 2.3. Screening and Data Extraction

We used the Nested Knowledge platform for screening and data extraction. Two people independently reviewed the titles and abstracts of all collected records, checking them against our set inclusion and exclusion rules. The two reviewers closely read the complete articles that appeared suitable to determine if they should be kept. Disagreements addressed by involving the third reviewer. One reviewer pulled data from the chosen studies, and another checked the work to ensure it was correct and consistent. We collected details like author, year, diagnosis, sample size, test specimen, comparison group, diagnostic metrics, AI model, reference standard, outcomes, findings, and conclusions. Everything was organized in a table for easier review.

To be more specific and transparent, the testing reference or ‘gold standard’ methods for ascertaining VRF presence/absence for each of the studies [13,14,16,17,20,21,22,23,24,25] abstracted are detailed in Table 1. These included testing on either stereomicroscopic examination/micro-CT analysis for ex vivo studies, and consensus diagnosis for retrospective or in vivo collections. The diagnostic metrics referenced were contextualized within reference standards.

### 2.4. Quality Assessment

The quality of studies included in this systematic review was assessed using the Quality Assessment of Diagnostic Accuracy Studies (QUADAS-2) tool, designed to evaluate diagnostic accuracy studies for risk of bias and applicability [26]. The tool’s four domains were systematically examined: patient selection, index test, reference standard, and flow and timing. The patient selection domain verified consecutive or random sampling for VRF cases, excluding biassed omissions. The index test and reference standard domains confirmed independent AI diagnostics and consistent VRF confirmation. Flow and timing ensured uniform reference use and proper timing, with each rated for bias and applicability to judge study quality.

**Figure 1:**
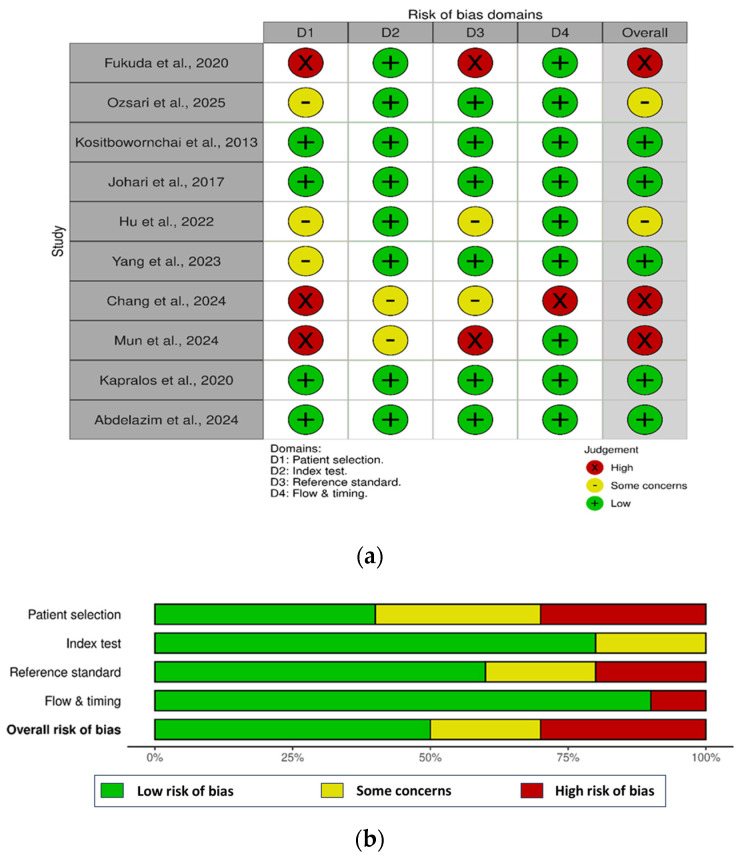
(**a**,**b**) Risk of bias assessment for included studies using QUADAS-2 domains [13,14,16,17,20,21,22,23,24,25].

The risk of bias assessment revealed varying quality across studies. Patient selection ranged from low to high risk, with some concerns noted. Index test was mostly low risk, with occasional moderate issues. Reference standard use was predominantly low risk, though some high-risk instances appeared. Flow and timing generally showed low risk, with a few high-risk cases. Overall bias risk spanned low to high, with Fukuda et al. (2020) [13] and Chang et al. (2024) [17] at high risk, while Kosibowornchai et al. (2013) [20] and Kapralos et al. (2020) [21] remained low risk (Figure 1).

High methodological and statistical heterogeneity among included studies precluded a quantitative meta-analysis of findings. The included studies were significantly heterogeneous in terms of the AI model type (CNN, PNN, and DNN), imaging modality (periapical, panoramic, CBCT), data preprocessing, and the nature of outcome re-porting for accuracy, sensitivity, specificity, AUC, and F1-score. Lack of uniformity of effect measures and comparable validation frameworks meant that statistical pooling could not be carried out. Consequently, the authors conducted a structured narrative synthesis of the included studies, based on the PRISMA 2020 recommendations.

## 3. Results

### 3.1. Literature Search and Selection

**Figure 2:**
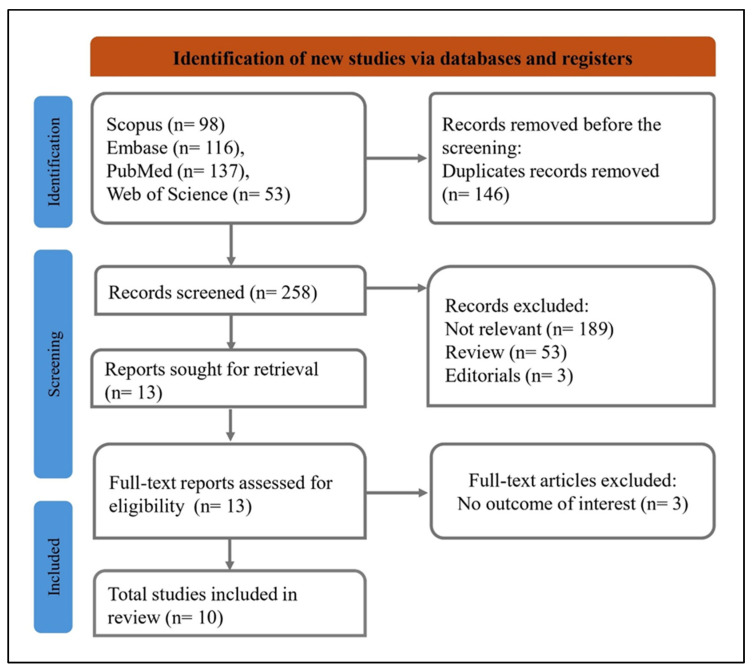
PRISMA flow diagram showing the study selection process in a systematic review.

We searched several databases—Scopus (98 records), Embase (116 records), PubMed (137 records), and Web of Science (53 records)—to find relevant studies (Figure 2). After eliminating 146 duplicates, we screened 258 records. From these, we excluded 245: 191 were irrelevant, 51 were review articles, and 3 were editorials. Then, we retrieved 13 full-text reports for a detailed review. Following eligibility evaluation, 3 articles were excluded due to the absence of relevant outcomes, resulting in 10 [13,14,16,17,20,21,22,23,24,25] studies included in the final review. This approach ensured a thorough and organized selection of studies that matched the research goal.

During the process of title and abstract screening, 18 out of 258 records (6.9%) caused discrepancies between the two main reviewers, and these discrepancies were resolved with a third reviewer. At the full-text article review stage, 2 out of 13 articles (15.4%) caused discrepancies and were sent for adjudication. All discrepancies were resolved via consensus without requiring exclusion due to disagreement among reviewers.

### 3.2. Overview of Included Studies

The studies, published up to July 2025, included different research types and settings, like hospital-based retrospective reviews, lab-based ex vivo tests, and patient data analyses. They used tools such as neural networks, statistical models, and combined methods, working with datasets of 120 to 418 radiographic images or teeth, and sample sizes from 200 extracted premolars to 552 hand-picked regions of interest. Images were adjusted with techniques like grayscale, normalization, cropping, resizing, wavelet changes, and data boosting. The imaging methods were intraoral digital radiography, panoramic radiography, CBCT, and periapical radiographs, all performed with standard equipment and settings.

Validation used methods like train-test splits, fivefold or 10-fold cross-validation, and measures like accuracy, sensitivity, specificity, precision, recall, F1-score, and AUC. Some studies also checked agreement between observers using kappa and intraclass correlation coefficients.

### 3.3. Performance of AI Models in Detecting Vertical Root Fractures Across Imaging Modalities

A total of 10 studies evaluated AI–based methods for detecting VRFs across various imaging modalities (Table 2). Convolutional neural network (CNN) architectures were the most frequently applied. Fukuda et al. (2020) [13] trained a CNN with DetectNet on 300 panoramic images, achieving a recall of 0.75, precision of 0.93, and F-measure of 0.83, with a VRF detection rate of 20% and 20 false positives. Hu et al. (2022) [16] reported higher performance using pretrained CNNs on CBCT images, with manual region-of-interest selection yielding an accuracy of 97.8% (AUC, 0.99) and automatic selection yielding 91.4% (AUC, 0.96). Abdelazim et al. (2024) [25] tested an ensemble of five pretrained CNNs on periapical radiographs, with DenseNet121 and DenseNet169 producing the best results (sensitivities, 89.4% and 87.2%; specificities, 93.6% each; AUC, 0.98).

Transfer learning–based hybrid CNNs were also investigated. Ozsari et al. (2025) [22] found that a DenseNet–Inception fusion with image enhancement on intraoral periapical radiographs achieved an accuracy of 0.80, precision of 0.81, recall of 0.80, F1-score of 0.80, and AUC of 0.80, with moderate-to-substantial interobserver agreement (κ = 0.60). Yang et al. (2023) [14] applied a fine-tuned ResNet-50 model to in vivo CBCT slices, achieving 94.5% sensitivity, 73.2% specificity, and an AUC of 0.929. Mun et al. (2024) [24] reported that among three models trained on panoramic radiographs, ResNet-50 had the highest sensitivity (90.43%) but lower specificity (60.77%), with an accuracy of 75.84% and AUC of 0.825.

Probabilistic neural networks (PNNs) were examined in 2 ex vivo studies. Kositbowornchai et al. (2013) [20] reported sensitivity of 97.2% to 98.0%, specificity of 60.0% to 90.5%, and accuracy of 88.3% to 95.7% depending on parameter settings. Johari et al. (2017) [23] found that for periapical images, a PNN achieved 97.78% sensitivity and 70.0% accuracy, whereas for CBCT, accuracy was 96.6% and specificity 100%, outperforming multilayer perceptron (MLP) models.

Other approaches included a deep neural network (DNN) compared with a support vector machine (SVM) in a retrospective CBCT study by Chang et al. (2024) [17], in which the DNN achieved 80.7% accuracy, 93.7% recall, and 86.7% F1-score, surpassing the SVM. Kapralos et al. (2020) [21] compared digital subtraction radiography (DSR) with conventional periapical radiography in root-filled teeth, reporting superior discrimination for DSR (AUC, 0.73 vs. lower for conventional).

Across studies, AI-assisted VRF detection demonstrated high sensitivity and specificity, particularly with CBCT-based CNN models or optimized ensemble approaches, though performance varied by imaging modality and study design.

### 3.4. Comparative Synthesis of AI Performance Across Modalities

A cross-study synthesis showed that there was a uniform trend for diagnostic accuracy based on imaging modality. The accuracy and specificity for CBCT-based AI models were the greatest (91.4–97.8% and 90.7–100%, respectively), followed by models based on periapical radiography images and then panoramic images. CNN-based architectures like DenseNet and ResNet were more superior. The designs were usually exaggerated due to optimal imaging conditions ex vivo, but realistic outcomes were obtained using in vivo datasets. The synthesis emphasizes that CBCT represents a gold standard for VRF diagnosis aided by AI.

### 3.5. Certainty of Evidence (GRADE Assessment)

A GRADE analysis was performed on CI-VRF using AI to determine the certainty of evidence. The quality of evidence was lowered for bias risk, particularly ex vivo and retrospective analyses, inconsistency among models, and indirectness because of data obtained from non-clinical sources. Based on these factors, it led to a classification of a certainty of evidence as moderate for AI systems based on CBCT images, low for periapical radiography images, and low for images obtained from panoramic radiography (Table 3).

## 4. Discussion

### 4.1. Overview of Findings

This systematic review compiled the existing evidence regarding the diagnostic accuracy of AI models in identifying VRFs using various imaging techniques, such as CBCT, periapical radiography, and panoramic radiography. Across the 10 included studies, AI approaches demonstrated considerable potential in assisting clinicians with VRF diagnosis, with performance varying according to the imaging modality, dataset characteristics, preprocessing techniques, and AI model architecture. Overall, CBCT-based AI systems consistently achieved the highest diagnostic metrics in vivo, while periapical radiography produced strong results in controlled ex vivo environments, and panoramic radiography showed comparatively lower diagnostic performance.

### 4.2. Comparison Across Imaging Modalities

The current findings align with earlier systematic reviews of AI in dental imaging, which have shown that 3D modalities such as CBCT tend to offer high diagnostic accuracy compared with 2D imaging, largely due to the reduction in anatomical superimposition and higher spatial resolution [27,28]. In the present review, Hu et al. [16] reported that fine-tuned convolutional neural networks (CNNs) applied to manually cropped CBCT regions of interest achieved an accuracy of 97.8%, sensitivity of 97.0%, and specificity of 98.5% (AUC 0.99), outperforming automatic selection workflows. Similarly, Johari et al. found that probabilistic neural networks (PNN) applied to CBCT images yielded an accuracy of 96.6% and perfect specificity, far exceeding the accuracy achieved using periapical radiographs in the same study (70.0%). Yang et al. [14] also demonstrated high sensitivity (94.5%) with CBCT slice-based CNNs, although specificity was slightly lower (73.2%), possibly reflecting the challenge of subtle fracture visualization in limited datasets.

In contrast, periapical radiography-based AI models showed greater variability in diagnostic performance. Abdelazim et al. [25] evaluated an ensemble of five pretrained CNNs on 2D periapical radiographs in an ex vivo setting, achieving up to 93.6% specificity and AUC values as high as 0.99 with DenseNet variants. Ozsari et al. [22] found that model fusion (DenseNet + Inception) with image enhancement yielded an accuracy of 80% and substantial interobserver agreement (kappa 0.60) in a clinical setting. However, these results, though promising, remain below the top-performing CBCT models, likely due to the inherent limitations of 2D projection imaging in detecting fractures masked by overlapping structures [29].

Panoramic radiography-based AI systems, while useful for screening, showed the lowest diagnostic performance. Fukuda et al. [13] reported that their CNN model with DetectNet architecture achieved an F-measure of 0.83 and a VRF detection rate of only 20% in a clinical dataset, with numerous false positives in non-fractured teeth. Mun et al. [24] used InceptionV3, ResNet50, and EfficientNetB0 architectures to classify cracked teeth (including VRFs) from panoramic radiographs, with accuracies ranging from 72.0% to 75.8% and AUC values around 0.80–0.825. These results are consistent with previous work indicating that panoramic radiography is inherently less sensitive for VRF detection due to lower spatial resolution and greater distortion [30].

It should be noted that Chang et al. (2024) [17] did not employ image-based inputs but rather a predictive model using clinical and procedural variables. Therefore, its findings provide contextual insight into complementary, non-imaging AI approaches rather than direct diagnostic image interpretation.

### 4.3. Influence of Study Design and Dataset Characteristics

Diagnostic performance was also influenced by study design (ex vivo vs. in vivo) and dataset size. Ex vivo studies often reported higher sensitivity and specificity due to controlled conditions, absence of confounding anatomical noise, and optimized imaging parameters. For example, Kositbowornchai et al. [20] achieved up to 98.0% sensitivity and 95.7% accuracy with a PNN model using extracted teeth imaged under standardized periapical protocols. Similarly, Abdelazim et al. [25] reported excellent metrics in an ex vivo setting with balanced datasets. However, these results may overestimate real-world performance, as clinical images are subject to patient movement, varied exposure parameters, and complex background anatomy [31].

Dataset size and diversity are critical for generalizable AI models [32]. Hu et al. [16] used a relatively large CBCT dataset (552 manually cropped ROIs and over 1000 auto-selected ROIs), which likely contributed to the high performance and robust validation results. In contrast, Yang et al. [14] used CBCT slices from only 14 patients, which, while achieving high sensitivity, may not represent the broader variability in fracture patterns. Limited datasets also increase the risk of overfitting, especially when using deep architectures with many trainable parameters [33].

### 4.4. Impact of Preprocessing and Model Architecture

Image preprocessing played a significant role in enhancing model performance. Studies employing targeted ROI cropping, image sharpening, augmentation, and feature extraction reported improved results. Hu et al. [16] demonstrated that manual cropping to tooth-level ROIs outperformed auto-selection, highlighting the value of precise localisation in VRF detection. Ozsari et al. [22] incorporated deep learning-based image enhancement and particle swarm optimization, which improved accuracy over baseline models.

Model architecture choice also influenced outcomes. DenseNet and ResNet variants, with their feature reuse and skip connections, were commonly associated with high AUC values across modalities [22,34,35]. Ensemble approaches, as seen in Abdelazim et al. [25], helped mitigate individual model weaknesses, resulting in higher overall diagnostic accuracy. In contrast, older architectures such as PNNs (used by Kositbowornchai et al. [20] and Johari et al. [23]) still performed competitively in smaller, well-controlled datasets, suggesting that simpler models may suffice when feature sets are limited and noise is low.

### 4.5. Comparison with Human Observers

Direct comparisons between AI models and human observers were limited but informative. Yang et al. [14] reported that their ResNet-50 CBCT model achieved higher sensitivity than two oral radiologists, though specificity was lower. Ozsari et al. [22] measured interobserver agreement (ICC) between AI and clinicians, finding moderate-to-substantial agreement, indicating that AI could serve as a useful adjunct to human diagnosis. These findings are consistent with broader radiology literature showing that AI can match or exceed human performance in specific tasks, especially when integrated into decision-support workflows [34,35].

### 4.6. Clinical Implications

The synthesized evidence from the included studies indicates that AI-assisted interpretation of CBCT images presently achieves the most consistent diagnostic accuracy for VRF detection. Across studies using convolutional neural-network models, CBCT-based AI reached accuracies of approximately 91–98% and specificities up to 100%, suggesting a clear diagnostic advantage under experimental or limited clinical conditions. Nevertheless, these results derive mainly from single-centre or ex vivo investigations with moderate-certainty evidence, and generalization to routine clinical use remains unproven.

AI models trained on periapical and panoramic radiographs showed variable but promising performance, generally accuracies ranged from 70 to 95%, especially when image-enhancement or ensemble methods were used. These findings support their potential as adjunctive or preliminary diagnostic tools rather than stand-alone systems. In practical terms, 2D AI-based analyses may contribute to early detection of potential fractures, while CBCT will still be the modality of confirmation in cases with lingering suspicion.

Although multiple studies addressed AI image processing and observer-support capabilities, none of the available evidence directly addressed workflow integration, clinical usability, or decision-support outcomes. Any implication of AI deployment in dental imaging workflows should thus be considered a priority for future research. Clinicians should interpret AI outputs with caution in light of small, non-standardized datasets, limited external validation, and possible false-positive outcomes resulting from anatomic complexity.

### 4.7. Limitations of the Evidence Base

The included studies varied considerably in design, dataset composition, image acquisition parameters, and reporting of metrics, limiting direct comparability. Many studies relied on retrospective data, and only a minority tested models prospectively in clinical settings. Few studies assessed external validity through testing on datasets from different institutions or imaging systems, which is critical for generalisability. Additionally, reporting standards for AI studies in dental imaging remain inconsistent, with several studies omitting confidence intervals, detailed preprocessing steps, or calibration plots.

The interpretation of diagnostic accuracy should also be made with consideration of inter-protocol variability with regard to reference standards. Although a reference standard may have included direct visualization/micro-CT verification in some ex vivo analyses, it included either radiographic consensus or surgical verification within clinical analysis. It might explain some variability in terms of accuracy and AUC values.

The fact that there is not a meta-analysis result represents an indication about the large variability within AI models, datasets, and reporting metrics among studies, so there is not a possibility to calculate a meaningful statistical aggregation of diagnostic performance metrics

Apart from methodological heterogeneity, there was low to moderate certainty of evidence based on the GRADE approach. There was moderate certainty of evidence for the use of CBCT-based AI systems because there were various consistent studies with reliable data. However, there was low certainty of evidence for periapical and panoramic models as there were small sample populations and inconsistencies on how they were validated.

### 4.8. Future Directions

Future research should prioritize multi-centre, prospective validation of AI models with large, diverse datasets representing a range of patient demographics, tooth types, and fracture morphologies. Developing modality-specific AI tools optimized for CBCT, periapical, and panoramic imaging could improve adoption in settings with differing resources. Moreover, combining image-based AI outputs with clinical data—such as history of trauma, occlusal load, and previous endodontic treatment—could yield hybrid models with superior diagnostic performance.

The application of explainable AI methods, including class activation mapping and attention visualization, should be expanded to enhance transparency and clinician trust. Finally, cost-effectiveness analyses are needed to determine the value proposition of integrating AI into dental imaging workflows relative to standard care.

Taken together, AI-assisted CBCT represents the most reliable method of diagnosis of VRFs, whereas the application of AI to 2D imaging modalities remains a developing adjunct to early screening. The field is moving from feasibility towards validation; future progress will be contingent upon robust, multicentre datasets, coupled with transparent model benchmarking to guarantee reproducible clinically interpretable AI deployment in endodontic diagnostics.

## 5. Conclusions

This systematic review critically appraised evidence on the use of AI for the detection of VRFs from CBCT, periapical, and panoramic imaging. Included studies consistently reported that CBCT-based AI models have the highest diagnostic accuracy and specificity, supported by a moderate certainty of evidence under controlled or single-institution conditions. Panoramic and periapical AI systems, though promising in their accuracy within experimental contexts, remain preliminary adjuncts whose real-world clinical performance needs to be verified.

Taken together, the strength of evidence drawn from the current literature is compromised by the presence of heterogeneous methodologies, small data sets, and a lack of external validation. Therefore, emphasis in future studies should be on multicentre prospective designs, standardized imaging protocols, and cross-modality comparative validation to enhance reproducibility and reliability. Only once this has been addressed can AI move from research studies to clinical dependency for diagnostic support for VRF detection.

## Figures and Tables

**Table 1:** GRADE Summary of Evidence for AI-Assisted Detection of Vertical Root Fractures (VRFs) Across Imaging Modalities.

Imaging Modality	No. of Studies (*n*)	Principal AI Architectures Evaluated	Representative Diagnostic Performance (Accuracy/Sensitivity/Specificity/AUC)	Risk of Bias	Inconsistency	Indirectness	Imprecision	Publication Bias	Overall Certainty of Evidence (GRADE)	Explanatory Notes
Cone-Beam Computed Tomography (CBCT)	3 (Yang et al., 2023 [14]; Hu et al., 2022 [16]; Johari et al., 2017 [23])	CNNs (ResNet-50, VGG19, DenseNet169) and PNN comparisons	91.4–97.8/93–97/90–100/AUC 0.96–0.99	◕◕◕○	◕◕◕○	◕◕◕◕	◕◕◕○	◕◕◕○	Moderate	Consistently high accuracy in in vivo datasets; minor bias from single-centre design and limited sample sizes.
Periapical Radiography	4 (Kositbowornchai et al., 2013 [20]; Ozsari et al., 2025 [22]; Johari et al., 2017 [23]; Abdelazim et al., 2024 [25])	PNN, CNN (DenseNet, VGG, Inception fusion)	70–95.7/87–98/67–94/AUC 0.74–0.99	◕◕○○	◕◕○○	◕◕◕○	◕◕○○	◕◕○○	Low	High performance in controlled ex vivo conditions but low clinical generalizability and heterogeneous reporting.
Panoramic Radiography	3 (Fukuda et al., 2020 [13]; Chang et al., 2024 [17]; Mun et al., 2024 [24])	CNN (DetectNet, ResNet-50, InceptionV3, EfficientNetB0); DNN vs. SVM	72–83/45–94/52–93/AUC ≈ 0.80–0.83	◕◕○○	◕◕○○	◕◕◕○	◕◕○○	◕◕○○	Low	Useful for screening applications but insufficient certainty for diagnostic decision-making due to limited resolution and bias.

GRADE symbols—◕◕◕◕ = no or very low concern; ◕◕◕○ = minor concern; ◕◕○○ = serious concern.

**Table 2:** Characteristics of included studies.

Author	Study Design/Setting	AI Model	Dataset Type and Size (*N*)	Preprocessing Techniques	Image Acquisition	Validation Method	Application	Outcomes
Fukuda et al., 2020 [13]	Retrospective model development/Clinical (hospital settings)	Convolutional Neural Network (CNN) with DetectNet	Panoramic images, 300 (330 VRF teeth, ~900 × 900 pixels)	Cropping to 900 × 900 pixels, labelling with rectangular regions of interest by oral radiologist	Panoramic radiography	Fivefold cross-validation	Diagnosis of VRFs	Recall (0.75), Precision (0.93), F-measure (0.83); 20% VRF detection rate, 20 false positives in non-fractured teeth
Ozsari et al., 2025 [22]	Experimental study with intraoral periapical radiographs	Transfer learning: DenseNet-121, ConvNext, InceptionV3, MobileNetV2, Model fusion	378 intraoral periapical radiographs (195 teeth with fractures, 183 without fractures)	DL-based image enhancement, Particle Swarm Optimization (PSO)	Intraoral periapical radiographs, digital CCD sensor (1.25 million pixels) with 20 lp/mm resolution, exposure time 0.1–0.4 s	Accuracy, precision, recall, F1, AUC, kappa, ICC for observer/model agreement, *t*-test for statistical significance, observer annotation	VRFs detection	DenseNet+ Inception+ enhancement: accuracy 0.80, precision 0.81, recall 0.80, F1 0.80, AUC 0.80, kappa 0.60; ICC moderate-substantial
Kositbowornchai et al., 2013 [20]	Ex vivo study; experimental design using extracted human teeth in laboratory conditions	Probabilistic Neural Network (PNN)	200 digital periapical images: 150 fractured roots, 50 intact roots; three groups with different test splits: Group 1: 80 train/120 test Group 2: 105 train/95 test Group 3: 130 train/70 test	Grey-scale data extracted from line scan across root image (40 points per line); 3 lines per root for training and 1 line for testing; normalization to reduce grey-scale variance	Intraoral digital radiographs using RVGui sensor, parallel technique, facio-lingual direction; 65 kVp, 8 mA, 0.08 s exposure	Separate training and test sets; groups tested at multiple Gaussian variance parameters (0–1)	Detection of VRFs on dental radiographs	Group 1: Sensitivity 97.8%, Specificity 60.0%, Accuracy 88.3%; Group 2: Sensitivity 97.2%, Specificity 78.3%, Accuracy 92.6%; Group 3 (130 train/70 test): Sensitivity 98.0%, Specificity 90.5%, Accuracy 95.7% (variance 0.025–0.005)
Johari et al., 2017 [23]	Ex vivo experimental	Probabilistic Neural Network (PNN), benchmarked against Multilayer Perceptron (MLP)	240 images (120 VRF, 120 non-VRF); periapical and CBCT radiographs	Daubechies 3 wavelet transform, Gabor filters (8 directions), image compression (160 × 120 to 16 × 12 pixels)	Periapical: Kodak RVG 5100 (60 kVp, 7 mA, 0.08 s); CBCT: NewTom VGi (110 kVp, 4.71 mA, 3.6 s, 1 mm slices)	Three data splits (varying train/test ratio); sigma tuning; comparison PNN vs. MLP	Detection of VRFs in premolars from periapical and CBCT images	Periapical radiographs: accuracy 70.0%, sensitivity 97.78%, specificity 67.7% CBCT: accuracy 96.6%, sensitivity 93.3%, specificity 100% PNN outperformed MLP in all metrics, but MLP had faster training
Hu et al., 2022 [16]	Retrospective in vivo study using CBCT images of patients’ teeth	Convolutional Neural Networks (CNNs): ResNet50, VGG19, DenseNet169 pretrained and fine-tuned on tooth ROIs	276 VRF teeth + 276 non-VRF teeth (total 552 ROIs) manually cropped; 1118 ROIs in auto-selection group	Sharpening, image augmentation (flip, rotation, brightness, contrast), ROI cropping (manual & auto)	CBCT: NewTom VG scanner, 0.15 mm voxel size, 110 kV, 3.6–3.7 mA, 12 × 8 cm FOV, 5.4 s acquisition time	Fivefold cross-validation, 75% training/25% testing split	Detection of VRFs in non-endodontically treated teeth on CBCT images in vivo	Manual selection group: Accuracy 97.8%, Sensitivity 97.0%, Specificity 98.5%, AUC 0.99; Auto-selection group: Accuracy 91.4%, Sensitivity 92.1%, Specificity 90.7%, AUC 0.96
Yang et al., 2023 [14]	Retrospective study using in vivo CBCT images from 14 patients	Convolutional Neural Network (ResNet-50) fine-tuned on CBCT slices	In vivo: 28 teeth (14 with VRF, 14 intact), 1641 CBCT slices	ROI cropping, resizing to 224 × 224 pixels, data augmentation, class activation mapping	CBCT NewTom VG, FOV 15 × 15 cm, voxel size 0.25 mm, 110 kVp, 1.60–4.30 mA, scan time 24 s	Data split into training/ validation/ test; comparison with two oral maxillofacial radiologists; ICC for interobserver agreement	Automated detection of VRF on CBCT slices from patient teeth	Sensitivity: 94.5% Specificity: 73.2% Accuracy: 83.9% AUC: 0.929
Chang et al., 2024 [17]	Retrospective clinical study using patient data	Deep Neural Network (DNN), compared with Support Vector Machine (SVM)	145 clinical teeth cases: 97 fractured teeth and 48 non-fractured teeth; 17 mixed-type features	Ordinal encoding for categorical features; min-max normalization	Not specified	5-fold cross-validation	Predicting the likelihood of VRF occurrence after root canal treatment	DNN: Accuracy 80.7%, Recall 93.7%, Precision 80.6%, F1-score 86.7%; SVM: Accuracy 71.7%, Recall 94.7%, Precision 71.9%, F1-score 81.7%
Mun et al., 2024 [24]	Retrospective clinical study	Deep learning image classification models: InceptionV3, ResNet50, EfficientNetB0	418 cropped individual tooth images (209 cracked including VRF subset, 209 normal)	Tooth polygon cropping, zero-padding to square, resizing to 224 × 224 pixels, pixel normalization (scaled 0–1)	Panoramic radiographs from PCH-2500 (Vatech) and Promax (Planmeca); 12-bit DICOM images, varying exposure parameters	Fivefold cross-validation with 3:1:1 split	Predict indication for cracked tooth extraction (including VRF) on panoramic radiographs using AI classification of individual teeth	InceptionV3: Sensitivity 94.26%, Specificity 52.63%, Accuracy 72.01%, F1-score 76.36%, AUC ~0.80; ResNet50: Sensitivity 90.43%, Specificity 60.77%, Accuracy 75.84%, F1-score 79.00%, AUC 0.825; EfficientNetB0: Sensitivity 92.54%, Specificity 54.23%, Accuracy 73.44%, F1-score 77.06%, AUC ~0.81
Kapralos et al., 2020 [21]	Ex vivo study	Used digital subtraction radiography (DSR) software	60 teeth; imaged at 0°, +10°, −10° angles; both CDPR and DSR images	Geometric registration and subtraction of pre- and post-fracture images	Digital periapical radiography (Gendex), 65 kV, 10 mA, 0.8 s exposure, parallel technique	5 expert evaluators	VRF detection with DSR vs. conventional radiography	Best (DSR, root-filled teeth): Sensitivity 0.45, Specificity 0.87, AUC 0.73; non-filled teeth AUC ~0.82–0.84; DSR better AUC than CDPR in filled teeth
Abdelazim et al., 2024 [25]	Ex vivo experimental study	Ensemble voting system combining five pretrained convolutional neural network (CNN) models: VGG16, VGG19, ResNet50, DenseNet121, DenseNet169	400 digital periapical radiographic images of extracted teeth, balanced 1:1 fractured vs. unfractured roots	Images resized uniformly to 224 × 224 × 3 pixels; normalized pixel values (scaled 1/255). No data augmentation applied to avoid possible overfitting or bias	Digital periapical images acquired with VistaScan system, processed with DBSWIN software	Training with 50 epochs per model; dropout 0.3 for regularization; early stopping (patience 15 epochs); reduce learning rate on plateau	Automated detection of root fractures in 2D periapical radiographs of extracted teeth using AI ensemble voting classifier to aid diagnostic accuracy	VGG16: Sensitivity (fractured) 87.0%, Specificity (fractured) 100%; Sensitivity (unfractured) 87.0%, Specificity (unfractured) 88.6%; ROC AUC 0.99; PPV fracture 93.3%, PPV unfractured 89.7%. VGG19: Sensitivity (fractured) 44.7%, Specificity (fractured) 74.5%; ROC AUC 0.74; PPV fracture 63.6%. ResNet50: Sensitivity (fractured) 42.6%, Specificity (fractured) 85.1%; ROC AUC 0.74; PPV fracture 59.7%. DenseNet121: Sensitivity (fractured) 89.4%, Specificity (fractured) 93.6%; ROC AUC 0.98; PPV fracture 93.3%, PPV unfractured 89.8%. DenseNet169: Sensitivity (fractured) 87.2%, Specificity (fractured) 93.6%; ROC AUC 0.98; PPV fracture 93.2%, PPV unfractured 88.0%

Note: Chang et al. (2024) [17] developed a DNN model using patient and treatment data to predict VRF risk post-endodontic therapy; not directly comparable to image-based AI models.

**Table 3:** Gold-Standard (Reference) Methods Used for Confirming the Presence or Absence of Vertical Root Fracture (VRF) in the Included Studies.

Author (Year)	Study Design/Setting	Imaging Modality Evaluated	Reference (Gold-Standard) Method for VRF Confirmation	Notes/Verification Level
Kositbowornchai et al., 2013 [20]	Ex vivo experimental	Periapical radiography	Direct visual inspection of extracted teeth after imaging under stereomicroscope	High-certainty physical confirmation of fracture lines
Johari et al., 2017 [23]	Ex vivo experimental	Periapical + CBCT	Micro-CT validation and stereomicroscopic visualization after sectioning	Dual-method verification; robust fracture confirmation
Fukuda et al., 2020 [13]	Retrospective clinical	Panoramic radiography	Consensus diagnosis by two experienced oral radiologists using combined clinical and radiographic data	Moderate-certainty consensus reference
Kapralos et al., 2020 [21]	Ex vivo experimental	Periapical radiography (DSR)	Visual inspection of induced fractures following imaging	High-certainty laboratory confirmation
Hu et al., 2022 [16]	Retrospective in vivo	CBCT	Expert consensus CBCT diagnosis by two endodontists, adjudicated by a third	Moderate-certainty radiologic reference
Yang et al., 2023 [14]	Retrospective in vivo	CBCT	Radiologist consensus diagnosis using validated CBCT reference set	Moderate-certainty radiologic reference
Chang et al., 2024 [17]	Retrospective clinical	CBCT (AI vs. SVM)	Clinical confirmation of VRF occurrence following root-canal treatment or surgical exploration	High-certainty operative confirmation
Mun et al., 2024 [24]	Retrospective clinical	Panoramic radiography	CBCT verification and chart-documented diagnosis	Moderate-certainty reference
Abdelazim et al., 2024 [25]	Ex vivo experimental	Periapical radiography	Direct visual inspection of extracted teeth after imaging	High-certainty physical confirmation
Ozsari et al., 2025 [22]	Retrospective clinical	Periapical radiography	Dual-radiologist consensus with third-party adjudication	Moderate-certainty consensus reference

## Data Availability

No new data were created or analyzed in this study. Data sharing is not applicable to this article.

## Supplementary Materials

The following supporting information can be downloaded at: https://www.mdpi.com/article/10.3390/diagnostics16030406/s1, Table S1: PRISMA checklist; Table S2: Inclusion and Exclusion criteria; Table S3: The adjusted search terms as per searched electronic databases (June 2025).

## Abbreviations

The following abbreviations are used in this manuscript:

AI	Artificial Intelligence
AUC	Area Under the Curve
CBCT	Cone-Beam Computed Tomography
CNN	Convolutional Neural Network
DNN	Deep Neural Network
DSR	Digital Subtraction Radiography
FOV	Field of View
ICC	Intraclass Correlation Coefficient
kVp	Peak Kilovoltage
mA	Milliampere
MLP	Multilayer Perceptron
PNN	Probabilistic Neural Network
PRISMA	Preferred Reporting Items for Systematic Reviews and Meta-Analyses
QUADAS-2	Quality Assessment of Diagnostic Accuracy Studies (Revised)
ROI	Region of Interest
SVM	Support Vector Machine
VRF/VRFs	Vertical Root Fracture/Vertical Root Fractures
